# Asepsis and Antiseptics

**Published:** 1909-10-02

**Authors:** 


### ASEPSIS AND ANTISEPTICS.

Dear Sir,—There is an article named "Aseptic Surgery" in the issue of The Hospital of September 11. To this article I must take exception. You say at the beginning "there is a steady increase in the popularity of aseptic as *opposed* to antiseptic surgery." Reading on, I see you laud the merits of heat as the new aseptic. Now what is heat but an antiseptic?—one of the best—so that there is no *opposition* at all between a- and anti-septic surgery. Surely antiseptics are just the agents we employ for attaining an aseptic condition?

I am yours truly,

General Practitioner.

Ayr, N.B., September 14.

[The late Professor Reclus somewhere defines the difference between aseptic and antiseptic surgery as the difference between peace and war. The definition is not scientifically accurate, but will serve for all practical purposes. By asepsis is meant scrupulous cleanliness—*i.e.* absence of micro-organisms, pathogenic or non-pathogenic, a condition analogous to what diplomats call a state of amity; by antisepsis is meant the killing of such micro-organisms—*i.e.* war. Heat is rarely used as an antiseptic except in cases where the actual cautery is employed.—Ed. The Hospital.]

